# ﻿Integrative taxonomy reveals overlooked cryptic diversity in the conifer feeding *Batrachedrapinicolella* (Zeller, 1839) (Lepidoptera, Batrachedridae)

**DOI:** 10.3897/zookeys.1085.76853

**Published:** 2022-02-08

**Authors:** Kai Berggren, Leif Aarvik, Peter Huemer, Kyung Min Lee, Marko Mutanen

**Affiliations:** 1 Bråvann terrasse 21, NO-4624 Kristiansand, Norway Unaffiliated Kristiansand Norway; 2 Natural History Museum, University of Oslo, P.O. Box 1172 Blindern, NO-0318 Oslo, Norway University of Oslo Oslo Norway; 3 Tiroler Landesmuseen Betriebgsges.m.b.H., Sammlungs- und Forschungszentrum, Naturwissenschaftliche Sammlungen, Krajnc-Str. 1, A-6060 Hall in Tirol, Austria Tiroler Landesmuseen Betriebgsges.m.b.H. Innsbruck Austria; 4 Zoology Unit, Finnish Museum of Natural History, University of Helsinki, Finland University of Helsinki Helsinki Finland; 5 Ecology and Genetics Research Unit, University of Oulu, Finland University of Oulu Oulu Finland

**Keywords:** Boreo-montane, ddRAD sequencing, DNA barcoding, Europe, Gelechioidea, new species, nuclear genes, Pinaceae

## Abstract

During efforts to generate DNA barcodes for all European Lepidoptera, *Batrachedrapinicolella* (Zeller, 1839) was found to comprise two genetically distinct clusters. Morphological investigation and results from two nuclear markers and ddRAD sequencing furthermore support the existence of two distinct taxa which we treat as two separate species, *B.pinicolella* and *B.confusella***sp. nov.** A lectotype for *B.pinicolella* is designated. Available data indicate that the biology of both species also differs, with *Piceaabies* (L.) Karsten as a proved host-plant for *B.pinicolella* and *Pinussylvestris* L. for *B.confusella***sp. nov.** Both species are mainly distributed on the European continent with *B.pinicolella* occurring in boreal parts of North and Central Europe and introduced to Canada, reflecting a boreo-montane distribution pattern. *Batrachedraconfusella***sp. nov.** is more widely distributed in temperate Northern and Central Europe.

## ﻿Introduction

During the last two decades, aided by DNA barcoding, several cryptic species have been discovered among European Lepidoptera (Huemer and Hebert 2011; Mutanen et al. 2012a, 2012b, 2020; Hernández-Roldán et al. 2016; Zlatkov and Huemer 2017, 2019; Huemer and Karsholt 2018; Huemer 2020; Wikström et al. 2020). The first suspicion that the widespread European pine feeding moth *Batrachedrapinicolella* (Zeller, 1839) consists of more than one species arose after the publication of the monograph of the European Momphidae s.l. (Koster and Sinev 2003). When the first author compared the genitalia figures on page 53 and 54 with the figures on page 253 and 319, discrepancies between these illustrations were observed. Independently within the framework of national barcoding initiatives in Norway and Finland as well as a supranational barcoding campaign in the Alps, we found striking genetic diversity in *B.pinicolella* indicating potential cryptic diversity (Huemer and Hebert 2016). Following this discovery, examination of morphological characters of representatives of the two clusters was made and additional molecular markers studied. Results confirm the presence in Europe of two separate species confused under the name *Batrachedrapinicolella*, one feeding on *Pinus* and the other on *Picea*.

## ﻿Material and methods

DNA barcodes refer to a 658 base-pair long fragment of the mitochondrial cytochrome c oxidase subunit 1 (CO1). Legs from 49 specimens of the involved species pair were prepared according to the prescribed standards and successfully processed at the Canadian Centre for DNA Barcoding (CCDB, Biodiversity Institute of Ontario, University of Guelph) to obtain DNA barcodes and using the standard high-throughput protocol described in deWaard et al. (2008). These sequences are supplemented by 16 DNA barcodes belonging to the two further known congeneric species of the European fauna. All sequences were submitted to GenBank, and further details including complete voucher data and images can be accessed in the public dataset “*Batrachedrapinicolella* species group [DS-BATRAPIN]” in the Barcode of Life Data Systems (BOLD; Ratnasingham and Hebert 2007). Degrees of intra- and interspecific variation of DNA barcode fragments were calculated under the Kimura 2 parameter model of nucleotide substitution using analytical tools of BOLD systems v. 4.0. (http://www.boldsystems.org). A neighbor-joining tree of DNA barcode data was constructed using MEGA 6 (Tamura et al. 2013) under the Kimura 2 parameter model for nucleotide substitutions.

We attempted to obtain data of six nuclear genes, wingless, *CAD* (carbamoyl-phosphate synthetase), *EF-1a* (elongation factor 1 alpha), *MDH* (malate dehydrogenase), *RpS5* (ribosomal protein S5), and *IDH* (isocitrate dehydrogenase), for specimens representing the two clusters of *B.pinicolella*. Five of these specimens represented BIN AAF0077 and three BIN AAF0078. Nuclear sequences were obtained for both clusters only for two of these genes, *EF-1a* and *MDH*. A few sequences of *CAD* and wingless were also recovered, but representing of only one of the BINs, and were therefore not considered in this study. We also studied the presence of *Wolbachia* bacteria in all eight specimens using *ftsZ* ja *wsp* markers (Zhou et al. 1998; Baldo et al. 2006). DNA extraction, PCR, and sequencing were conducted at the university of Oulu using standard Sanger sequencing protocols as outlined in Wahlberg and Wheat (2008), with slight modifications for example for purification of sequencing reactions. Sequencing was performed with an ABI 3730 capillary sequencer. Nuclear sequences were deposited in the Voseq database (Peña and Malm 2012). Genetic distances were calculated under Kimura 2 parameter model in MEGA 6 (Tamura et al. 2013). Nuclear sequences are available in GenBank under the accession numbers OM296685–OM296699. Genetic divergences between all four European species of *Batrachedra* were visualized with a neighbor-joining tree as conducted under Kimura 2 parameter model in MEGA 6 (Tamura et al. 2013).

We used DNA aliquots that were extracted at CCDB for ddRAD-seq library preparation. The quantity of DNA extracts was checked using PicoGreen kit (Molecular Probes). The ddRAD library was implemented following protocols in Lee et al. (2018) with few modifications: digestion with *Pst*I and *Msp*I, and the size distribution measurement with Bioanalyzer (Agilent Technologies). The de-multiplexed fastq data are archived in the NCBI SRA: PRJNA725165. Initial filtering steps, SNP calling, and alignment were carried out using *ipyrad* v.0.9.11 (Eaton and Overcast 2020). The following parameters were changed from the default settings: restriction overhang to *TGCAG*, *CGG*, minimum depth for majority-rule base calling to 3, clustering threshold to 0.88, and minimum number of samples with a given locus to 3.

To infer maximum likelihood (ML) tree, we used IQ-TREE (Nguyen et al. 2015), and branch support bootstrap values were calculated with ultrafast bootstrap (UFBoot, 1000 bootstraps). Prior to analyses, the best fitting mutational model was obtained through ModelFinder (Kalyaanamoorthy et al. 2017) based on Bayesian information criterion, which selected “TVM+F+I” for ddRAD data. The ML tree generated using FigTree v.1.4.2 (Rambaut 2015) and modified using Adobe Illustrator CS6. To investigate genetic variation between individuals, we inferred population clustering with admixture from SNP frequency data using STRUCTURE (Pritchard et al. 2000). Ten replicates were run at each value of K between 1 and 3. Each run had a burn-in of 50K generations followed by 500K generations of sampling. We used StrAuto to automate Structure processing of samples (Chhatre and Emerson 2017). Replicates were permuted in CLUMPP (Jakobsson and Rosenberg 2007) according to the ad hoc ∆K statistics (Evanno et al. 2005), and the results were visualised using DISTRUCT (Rosenberg 2004).

Dissections of genitalia followed Robinson (1976). Photos of genitalia were taken through a Leica DM 6000B microscope using a Leica DFC 420 digital camera.

### ﻿Depository of examined material

**KBE** Collection of Kai Berggren, Kristiansand, Norway

**NHMO** Natural History Museum, University of Oslo, Norway

**NHMUK** The Natural History Museum, London, U.K.

**TLMF** Tiroler Landesmuseum Ferdinandeum, Innsbruck, Austria

**ZMUC** Zoological Museum, Natural History Museum of Denmark, Copenhagen, Denmark

**ZMUO** Zoological Museum, University of Oulu, Finland

### ﻿Type material and nomenclature

**Figures 1–4.**
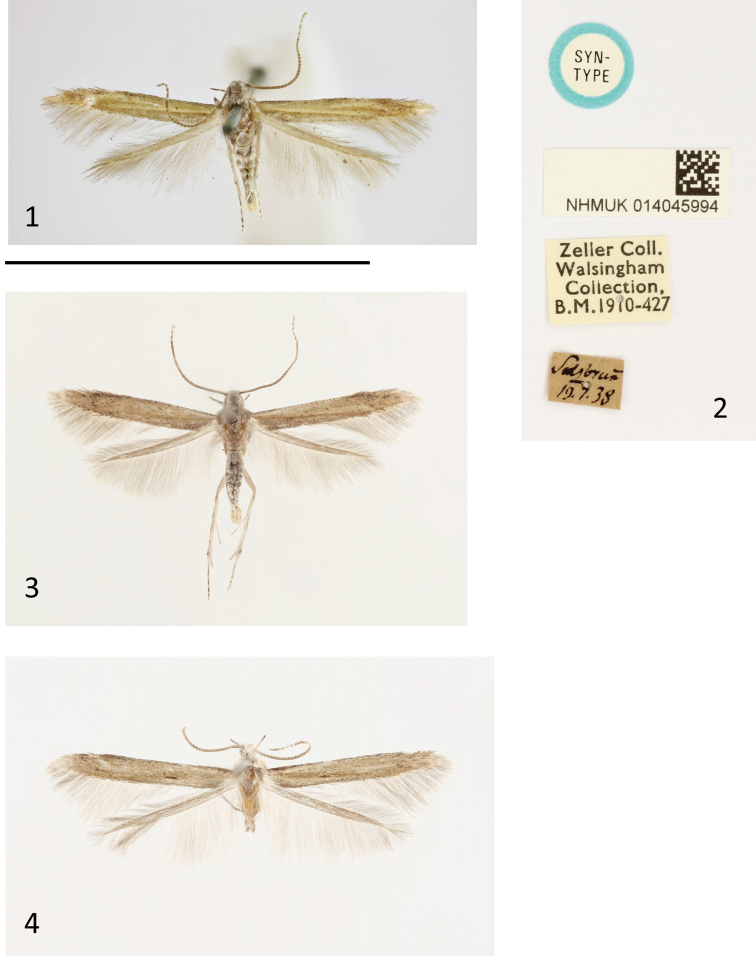
Adults and labels of *Batrachedra***1** lectotype *Cosmopteryxpinicolella* Zeller, 1839 (NHMUK; copyright Trustees of the Natural History Museum, London) **2** labels of lectotype **3** holotype of *Batrachedraconfusella* sp. nov. **4** adult of *Batrachedrapinicolella*. Scale: 10 mm.

Zeller (1839) described *Cosmopteryxpinicolella* from a series collected from *Pinus* near Glogau, “häufig bei Gl. im Juni und Juli an Kiefern” [common near Gl. = Glogau in June and July on pine], and another series collected near Salzbrunn, “noch häufiger bei Salzbrunn an Tannen” [more common near [Bad] Salzbrunn on fir]. Glogau (now Glógow) is situated in the south-western present-day Poland, and Bad Salzbrunn (now Szczawno-Zdrój) in Silesia (Poland) near the Czech border. Two potential syntypes of *pinicolella* are present in NHMUK where the collection of Zeller is preserved (David Lees, email 29 August 2019). One is without abdomen and has no locality label attached to it, and thus syntype status is uncertain. The other specimen is a male in good condition, labelled “Salzbrunn 19.7.[18]38”. We select the male from Salzbrunn as lectotype (Fig. 1) in order to fix the identity of the species and conserve stability of nomenclature. Though we have not been able to dissect this specimen, it can be identified without any doubt. The reasons for this are that it has a definite (type) locality and an indication to the alleged host tree. Zeller (1839) stated to have collected syntypes from Salzbrunn on “Tannen”. In Zeller’s work from 1839 “Tanne” (*Abiesalba* in current sense) is mentioned several times, whereas “Fichte” (*Piceaabies*) is completely missing. Even species considered monophagous on “Fichte”, such as *Chionodeselectella* (Zeller, 1839), are attributed to “Tanne”. Our hypothesis is that “Tanne” sensu Zeller (1839) is identical with *Piceaabies*. This is also supported by, for example, the later description of *Pammeneochsenheimeriana* (Lienig & Zeller, 1846) (known from *Picea* and *Pinus* but not from *Abies*). Also, in this description it is stated “im Mai auf Tannen” (Lienig and Zeller 1846). In the German language *Picea* is also called “Rottanne” and *Abies* “Weißtanne”. Our conclusion is that the name *B.pinicolella* (Zeller, 1839) must be attributed to the *Picea*-feeder and not to the *Pinus*-feeder, whereas *Abiesalba* seems an exceptional hostplant of this group with only one proven record of four larva collected in Slovakia (Patočka 1960). We are aware that attribution of the name *B.pinicolella* to the *Picea*-feeding taxon may cause confusion, but one should bear in mind that at the time of the description of *B.pinicolella* conifer trees currently in *Pinus*, *Picea*, *Abies*, and even *Larix* were all listed in one genus, *Pinus* (Hausmann 1852). Thus, the name *pinicolella* refers to the family Pinaceae rather than to the present genus *Pinus*. Furthermore, even in current European literature the name *B.pinicolella* is sometimes already expressively combined with the species from *Piceaabies* whereas *Pinussylvestris* is considered as doubtful hostplant which requires confirmation (Emmet and Langmaid 2002). Following the selection of a lectotype from *Piceaabies* there is no synonym of *B.pinicolella* that could potentially be used for the species feeding on *Pinussylvestris* (Koster and Sinev 2003) and, hence, this species is without name.

## ﻿Results

### ﻿ Batrachedra confusella sp. nov.

Taxon classificationAnimaliaLepidopteraBatrachedridae

586F763C-5AE2-5079-899A-AC35AF2A49EF

http://zoobank.org/AE37C32C-7D9A-4436-AAB2-339F486FF239

Figs 3 , 5 , 6 , 9

#### Material.

***Holotype*** 1♂ Ø, Moss: Rygge, Sildebauen, 59.3268°N; 10.7101°E; 9.vii.1980; L. Aarvik leg.; NHMO prep. 3943 (NHMO). ***Paratypes*** Finland 1♂ A, Sund; 14.vii.2007; M. Mutanen leg.; L. Aarvik prep. 2015.022; BOLD sample ID: MM22065; ZMUO. 1♂ PPe, Oulu; 7.vii.2011; M. Mutanen leg.; L. Aarvik prep. 2015.023; BOLD sample ID: MM21051; ZMUO. 1♀ U, Kirkkonummi; 11.vii.2007; M. Mutanen leg.; L. Aarvik prep. 2015.025; BOLD sample ID: MM06674; ZMUO.

Norway 1♂ AAY, Arendal: Havsøy; 10.vii.2015; K. Berggren leg.; BOLD sample ID: NHMO-DAR-12592; KBE. 1♀ AAY, Arendal: Tromøy, Skottjern; 22.vii.1983; K. Berggren leg.; KBE prep. 13534; KBE; 1♂, same locality and date; S. Svendsen leg.; NHMO prep. 2833; NHMO. 1♂ AAY, Arendal: Siring; 25–30.vi.2002; S.A. Bakke leg., NHMO prep. 3936; NHMO. 1♀ AAY, Grimstad: Søm; 8.vii.2017; K. Berggren leg., BOLD sample ID: NHMO-DAR-13683; KBE. 1♀ AAY, Grimstad: Søm; 24.vii.2017; K. Berggren leg.; BOLD sample ID: NHMO-DAR-14041; KB. 1♂ AAY, Lillesand: Lillesand; 7.vii.1984; K. Berggren leg.; KBE prep. 13515; KBE. 1♂ AAY, Lillesand: Svinøya; 29.vii.2005, K. Berggren leg.; KBE prep. 13520; KBE. 1♂ AAY Tvedestrand: Lyngør, Sønnerstrand; 30.vi–4.vii.2013; K. Berggren & B. Johansen leg.; KBE prep. 13524; KBE. 1♂ AK, Asker: Brønnøya; 11.vii.1981; K. Berggren leg.; KBE prep. 13528; KBE. 1♀ AK, Bærum: Ostøya; 10.vii.1984; L. Aarvik leg.; NHMO prep. 2887; NHMO. 1♂ AK, Oslo: Bleikøya; 14.vii.2009; K. Berggren & A. Endrestøl leg.; BOLD sample ID: NHMO-DAR-5192; KBE. 1♀ AK, Oslo: Bygdøy, Rodeløkka; 4–19.vii.2016; A. Endrestøl & K. Berggren leg.; KBE prep. 13536; KBE. 1♂ AK, Oslo: Bygdøy, Rodeløkka; 4–19.vii.2016; A. Endrestøl & K. Berggren leg.; KBE prep. 13532; KBE. 1♂ AK, Oslo: Ekebergskråninga; 25.vi.2008; L. Aarvik leg.; NHMO prep. 2835; BOLD sample ID: NHMO-08107; NHMO. 1♀ AK, Ås: Ås; 30.vii.1982; L. Aarvik leg.; L. Aarvik prep. 2725; NHMO. 1♀ HES, Åsnes: Sønsterud; 16–25.vii.1998; L. Aarvik & A. Bakke leg.; NHMO prep. 2832; NHMO. 1♀ TEY, Kragerø: Jomfruland, Øytangen; 16.vii.2003; L. Aarvik leg.; NHMO prep. 3937; NHMO. 1♂ TEY, Porsgrunn: Sandøya; 10.vii.2003; R. Voith leg.; KBE prep. 13521; KBE. 1♂ VAY, Kristiansand: Augland; 8.vii.1985; K. Berggren leg.; KBE prep. 13529; KBE. 1♂ VAY, Kristiansand: Bråvann; 14.vii.2015; K. Berggren leg., BOLD sample ID: NHMO-DAR-12591; KBE. 1♂ VAY, Kristiansand: Bråvann; 17.vii.2017; K. Berggren leg., BOLD sample ID: NHMO-DAR-14027; KBE. 1♀ VAY, Kristiansand: Bråvann; 10.viii.2012; K. Berggren leg.; KBE prep. 13537; KBE. 1♀ VAY, Kristiansand: Bråvann; 26.viii.2016; K. Berggren leg.; KBE prep. 13538; KBE. 1♂ VAY, Kristiansand: Bråvann; 26.viii.2016; K. Berggren leg.; BOLD sample ID: NHMO-DAR-12591; KBE prep. 13538; KBE. 1♂ VAY, Kristiansand: Flekkerøy, Belteviga; 28.vii.1999, K. Berggren leg.; KBE prep. 6141; KBE. 1♀ VAY, Kristiansand: Flekkerøy, Belteviga; 16–23.vii.2000, K. Berggren leg.; KBE prep. 6196; KBE. 1♀ VAY, Kristiansand: Nedre Timenes; 7–14.vii.2001, K. Berggren leg.; KBE prep. 101696; KBE. 1♂ VAY, Kristiansand: Nedre Timenes; 28.vii.2015, K. Berggren leg.; KBE prep. 13533; KBE. 1♀ VAY, Kristiansand: Nedre Timenes; 31.vii.2017, K. Berggren leg.; KBE prep. 13535; KBE. 1♂ VAY, Kristiansand: Østre Randøy; 9.vi.2006, K. Berggren leg.; KBE prep. 13530; KBE. 1♂ VAY, Kristiansand: Skålevik; 12.vii.2019; K. Berggren leg., BOLD sample ID: KBE-2019077; KBE. 1♂ VAY, Kristiansand: Stangenes; 4.vii.1981; S. Svendsen leg.; NHMO prep. 3899; NHMO. 1♀ VAY, Kristiansand: Ådnevik, Unndalen; 6.vii.2012; K. Berggren leg.; KBE prep. 13512; KBE. 1♀ VAY, Mandal: Hoven; 24–27.vii.2017; K. Berggren & K. Hoven leg.; KBE prep. 13513; KBE. 1♂ VAY, Mandal: Hoven; 22–24.vii.2017; K. Berggren & K. Hoven leg.; KBE prep. 13516; KBE. 1♂ VE, Horten: Knutsrød; 20–21.vi.2008; L. Aarvik leg.; NHMO prep. 3898; NHMO. 1♀ VE, Nøtterøy: Østre Bolærne; 27.vii.2006; R. Voith & K. Berggren leg.; KBE prep. 6104; KBE. 1♂ VE, Nøtterøy: Østre Bolærne; 9.vii.2006; R. Voith & K. Berggren leg.; KBE prep. 10171; KBE. 1♀ VE, Tønsberg: Karlsvikodden; vii.2005; R. Voith leg.; KBE prep. 10170; KBE. 1♂ Ø, Fredrikstad: Blåsopp; 4–19.vii.2013; O.J. Lønnve leg.; KBE prep. 13531; KBE. 1♂ Ø, Halden: Orød Grustak; 5.vi–1.vii.2009, F. Ødegaard leg.; BOLD sample ID: NHMO-DAR-4107 (failed); KBE prep. 9438; KBE. 1♂ Ø, Hvaler: Asmaløy, Huser; 3.vii.1994; L. Aarvik leg.; NHMO prep. 3965; NHMO. 1♂ Ø, Hvaler: Vesterøy, Slettevika; 25.vi.2018; J.R. Gustad leg.; NHMO prep. 3980; NHMO. 1♂ Ø, Moss: Rygge, Sildebauen; 13.vii.1980; L. Aarvik leg.; NHMO prep. 3890; NHMO. 1♂ Ø, Sarpsborg: Tune, Råkil; 4.vii.2002; T.J. Olsen leg., NHMO prep. 3935; NHMO.

Sweden 1♂ Öland, Borgholm: Byrums Sandvik; 23.vii.1985; K. Berggren leg.; KBE prep. 13527; KBE. 1♂ Öland, Borgholm: Byrums Sandvik; 23.vii.1985; K. Berggren leg.; KBE prep. 6142; KBE.

Denmark 1♂ EJ, Anholt; 1.viii.1975; E. S. Nielsen leg., Lundquist prep. 2050; ZMUC. 1♀ F, Æbelø; 25.vi.-9.vii.1943; Worm-Hansen leg.; Rasmussen prep. 3936; ZMUC. 1♂ NWZ, Kåruphøj; 18.vii.2003; H. Hendriksen leg.; Hendriksen prep. 4194; ZMUC. 1♂ LFM, Bøtø; 29.vi.1968; E. Traugott-Olsen leg.; Traugott-Olsen prep. 1242; ZMUC. 1♂, 1♀ LFM, Vålse Skov; 10.vii.1982; J. Lundqvist leg.; Lundqvist prep. 1343, 1344; ZMUC. 1♀ LFM, Ulfshale; 19.vii.1987; O. Karsholt leg.; Karsholt prep. 5381; ZMUC. 1♀ B, Slotslyngen; 31.vii.1967; H.K. Jensen leg.; Jensen prep. 692; ZMUC. 1♀ B, Boderne; 8.viii.1968; H.K. Jensen leg., Jensen prep. 810; ZMUC.

Austria 1♀, Oberösterreich, NP Kalkalpen, Spering-Lackerbodenstraße, ca 700 m; 16.vii.2004; J. Wimmer leg; genitalia in glycerine; TLMF. 1♀, ditto, but 653 m, 14.vi.2009; J. Wimmer leg; gen. slide GEL 1287 P. Huemer; TLMF. 1♂ Nordtirol, Umhausen N, unterh. Farst, 1370 m, 27.vi.2017, P. Huemer leg; GEL 1276 P. Huemer; TLMF. 1♂ Nordtirol, Umhausen N, unterh. Farst, 1370 m, 11.vi.2017, P. Huemer leg; DNA Barcode ID TLMF Lep 26819; TLMF. 1♂ Nordtirol, Zirl, Eigenhofen, linke Innau, 600 m, 7.vi.2012, P. Huemer leg.; DNA Barcode ID TLMF Lep 09322; TLMF. 1♂ Nordtirol, Brandenberg, Tiefenbachklamm, 645 m, 16.vi.2013, P. Huemer leg.; DNA Barcode ID TLMF Lep 10349; TLMF. 1♂ Osttirol, Lengberg, Drau-Auen, Schattseite, 630 m, 25.vi.2008, H. Deutsch leg.; DNA Barcode ID TLMF Lep 24185; TLMF. 1♂ Vorarlberg, Frödischtal, Schönebuchweg, Klausen, 755 m, 14.vi.2017, A. Mayr leg; DNA Barcode ID TLMF Lep 25068; coll. A. Mayr.

Germany 1♀, Bavaria, Inning/A, 550 m, mid vii.1970 Zürnbauer F. leg.; genitalia in glycerin PH; TLMF.

Italy 1♂ Südtirol, Oberrasen, Biotop Rasner Möser S, 1100 m, 5.vii.2015, P. Huemer leg.; DNA Barcode ID TLMF Lep 17982; TLMF. 1♂ Südtirol, Laas, Tschenglser Au, Biotop Rasner Möser S, 900 m, 20.vi.2015, P. Huemer leg.; DNA Barcode ID TLMF Lep 18779; TLMF.

**Figures 5–8.**
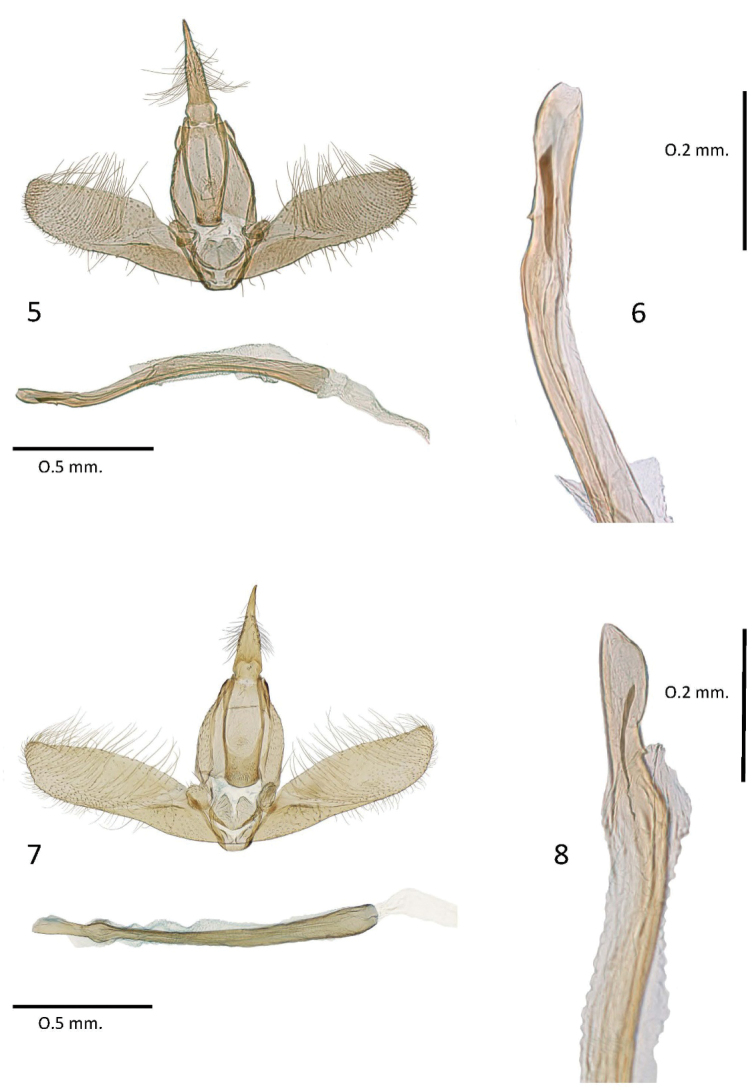
Male genitalia of *Batrachedra***5***B.confusella* sp. nov., genitalia slide NHMO 3899 **6***B.confusella* sp. nov., distal end of phallus, genitalia slide NHMO 3899 **7***B.pinicolella*, genitalia slide NHMO 3891 **8***B.pinicolella*, distal end of phallus, genitalia slide NHMO 3892.

Armenia 1♂, Tavush Province, Diljan, 1395 m; 13.vii.2011; O. Karsholt leg.; BOLD sample ID: ZMUC00029754; ZMUC.

#### Morphological diagnosis.

**Figures 9–10.**
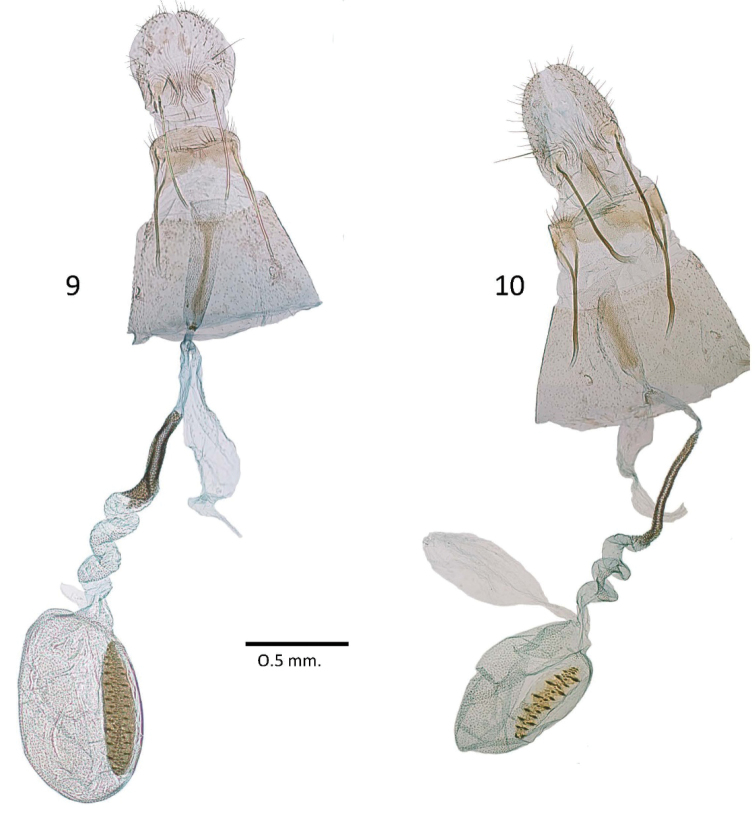
Female genitalia of *Batrachedra***9***B.confusella* sp. nov., genitalia slide NHMO 3937 **10***B.pinicolella*, genitalia slide NHMO 3961.

*Batrachedraconfusella* sp. nov. (Fig. 3) and *B.pinicolella* (Fig. 4) cannot be separated externally with certainty though it is remarkable that none of the 56 specimens of *B.confusella* with images in BOLD has a spot at about the one-third the length of the forewing fold, a character commonly present in *B.pinicolella*. All 47 specimens of Norwegian dissected or barcoded specimens of *B.confusella* sp. nov. are without the spot on the fold. In eight of 20 dissected or barcoded Norwegian specimens of *B.pinicolella* the spot is present. Thus, the presence of the spot in the fold strongly indicates that the actual specimen belongs to *B.pinicolella*. With a wingspan of 11.0–12.0 mm, *B.pinicolella* is slightly larger than *B.confusella* sp. nov. on average. In the male genitalia we found a diagnostic character in the shape of the valva and the uncus. On average *B.pinicolella* (Fig. 7) has a longer and more slender valva than *B.confusella* sp. nov. (Fig. 5). The uncus is slightly different in the two species in shape and width. In *B.confusella* sp. nov. the lateral sides are smoother and with finer and longer setae than in *B.pinicolella*. In *B.pinicolella* the uncus is laterally distinctly rugose with coarse setae. The tip of the phallus in *B.confusella* sp. nov. (Fig. 6) is armed with a distinct cornutus. In *B.pinicolella* (Fig. 8) the cornutus is narrower and often will appear merely as a fold inside the phallus. The gnathos tends to be broader distally in *B.pinicolella* than in *B.confusella* sp. nov. In the female genitalia, the two species differ in the length of the signum. In *B.pinicolella* (Fig. 10) the signum is in the range 290–380 μm. In *B.confusella* sp. nov. (Fig. 9) the length of the signum ranges from 490 to 600 μm. In *B.pinicolella* the sclerotized portion of the ductus bursae is narrower and straighter than in *B.confusella* sp. nov. Fig. 22 of the female genitalia given by Koster and Sinev (2003) represents *B.pinicolella* in the present interpretation of the name.

#### Molecular diagnosis.

**Figure 11.**
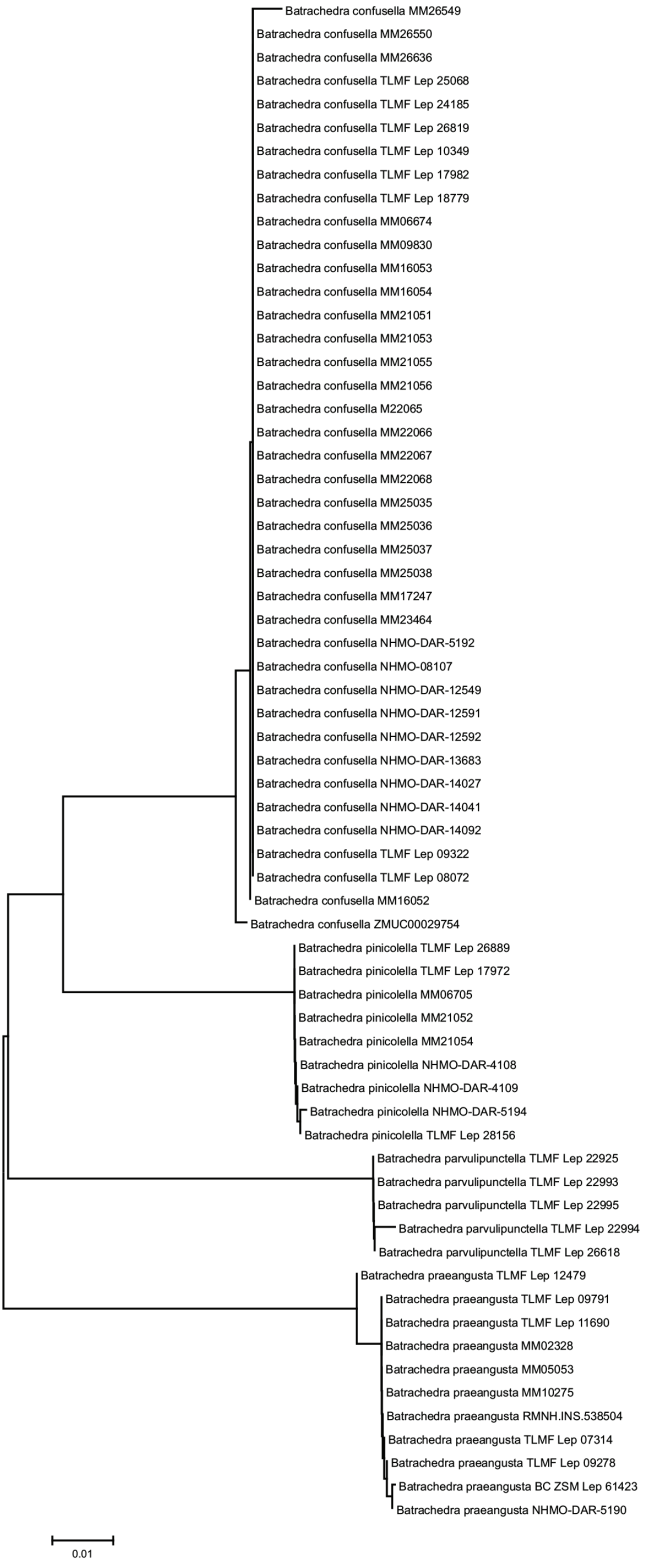
Neighbor-joining visualization of genetic divergences of DNA barcode fragment of COI gene within and between European species of *Batrachedra*. The scale indicates 1% genetic divergence as calculated under Kimura 2 parameter model for nucleotide substitution.

Sequences of *B.pinicolella* and *B.confusella* sp. nov. form two well-defined clusters which received separate Barcode Index Numbers (BIN-codes): AAF0078 and AAF0077 respectively. The DNA barcode region of *B.confusella* sp. nov. shows 0.95% intraspecific divergence and a minimum divergence of 6.82% to its closest relative *B.pinicolella* (Fig. 11). The latter species shows no intraspecific variability in our material (the slight apparent variation in the neighbor-joining tree results only from variation in sequence lengths). Therefore, DNA barcodes allow safe identification of the two species. The 407-bp long fragment of the nuclear MDH gene differs by 5.8% from that of *B.pinicolella*. The 506-bp long fragment of EF-1a differs by 3.1% from that of *B.pinicolella*. Therefore, both examined nuclear genes support the status of the two species and permit their identification by these markers as well. All examined five specimens were infected by *Wolbachia*, while none of the specimens of *B.pinicolella* were infected, suggesting a difference between the two species also in this regard.

**Figure 12.**
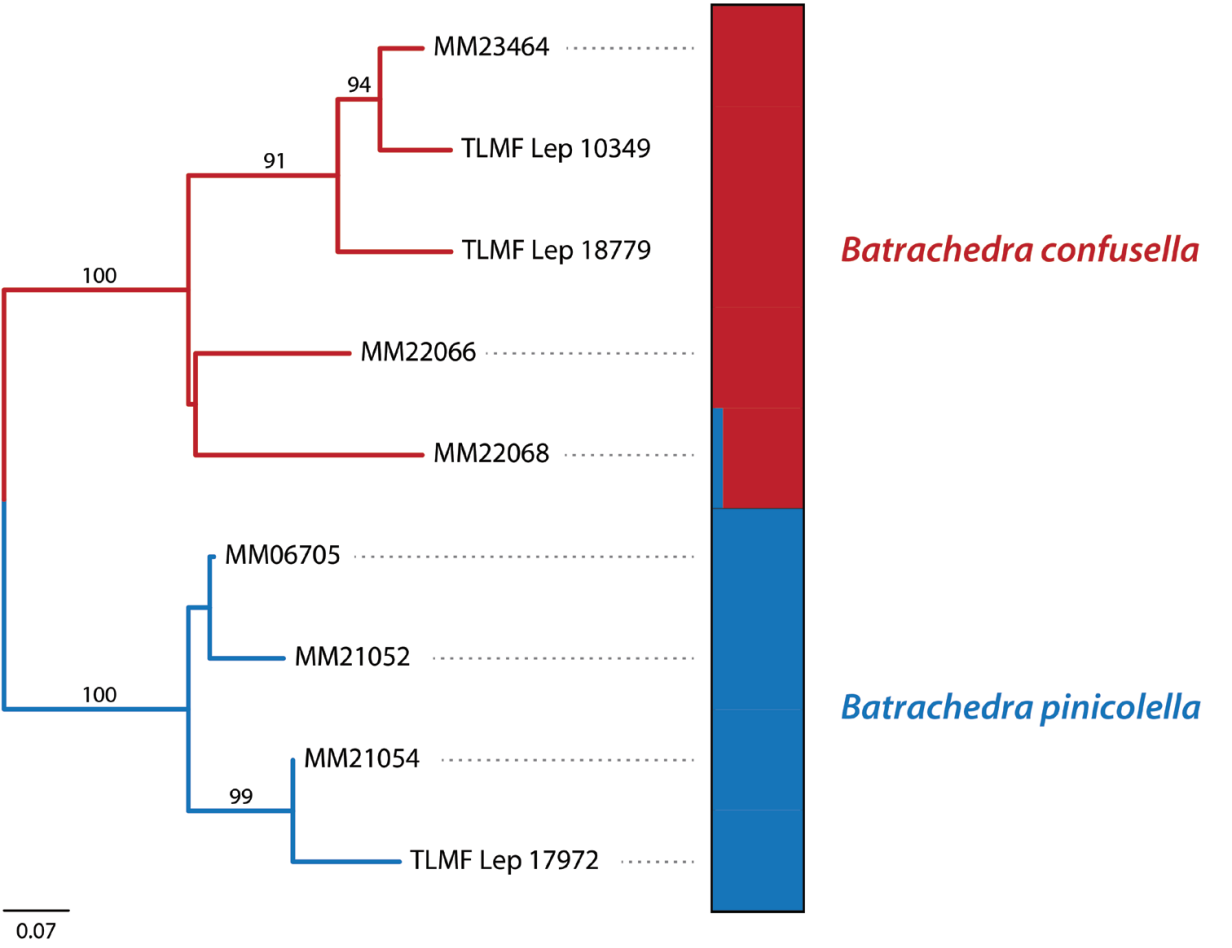
Maximum-likelihood tree inferred from the ddRAD SNP data. Bootstrap support values are indicated above the branches and only the values > 50% are shown. The barplot shows the assignments of individuals into two genetic clusters, the red clusters referring to *Batrachedraconfusella*, the blue clusters to *B.pinicolella*. Each bar represents one individual and colours represent the proportion of the individuals that belong to each of the genetic cluster.

We generated a genomic dataset from nine individuals of *B.pinicolella* using ddRAD sequencing. We obtained 1.24 million reads per individual on average after quality filtering steps. After clustering 88% sequence similarity, we recovered 19,292 clusters per sample were retained with an average of 82.26 per sample for cluster depth (Table 1). A total length of ddRAD data is 926,224 bp, of which 9,242 are single nucleotide polymorphisms (SNP). Phylogenetic analysis using SNP data produced robust support for the relationship between the individuals (Fig. 12). The two revealed lineages corresponding to *B.confusella* sp. nov. and *B.pinicolella*, which have 100% bootstrap support. STRUCTURE also identified two genetic clusters (Fig. 11).

**Table 1.** A summary of the ddRAD data.

Species	SampleID	Reads passed filter	Clusters at 88%	Coverage	Retained loci	Consensus loci
*B.confusella*	MM22066	1520904	29400	40.06	11608	4488
*B.confusella*	MM22068	1624893	31776	45.71	12030	4346
*B.confusella*	MM23464	2976135	62919	43.18	19568	4557
*B.confusella*	TLMF Lep 10349	1353160	16809	60.85	5292	2563
*B.confusella*	TLMF Lep 18779	624001	11396	47.99	3177	917
*B.pinicolella*	MM06705	808698	4251	163.44	572	149
*B.pinicolella*	MM21052	971904	6440	131.25	1305	200
*B.pinicolella*	MM21054	931540	7060	118.22	1344	370
*B.pinicolella*	TLMF Lep 17972	370227	3579	89.61	611	155
**Total**		**1242385**	**19292**	**82.26**	**6167**	**1972**

#### Description.

Male (Fig. 3). Wingspan 10–11 mm. Labial palp 2.3 times diameter of eye, cream, porrect, slightly curved, second segment longer than third, outer side with brownish suffusion, third segment on outer side with two bands. Head cream, with appressed scaling. Antenna glabrous, with appressed scales, pale ochreous brown, weakly ringed, rings becoming more distinct distally. Thorax ochreous yellow. Forewing ochreous yellow, with scattered dark brown scales, denser distally and on costa, discal spot small; cilia brownish grey; hindwing grey, cilia grey. Legs cream, inner sides with brownish suffusion, which on tarsi forms bands. Abdomen cream, with lateral grey suffusion.

**Female**. Externally similar to male.

**Male** genitalia (Figs 5, 6) Uncus gradually narrowed towards tip, slightly concave at two-thirds length, medially with lateral setae, lateral margins smooth, with fine hairs; gnathos gradually narrowed, becoming parallel-sided before distal end, distal end spinose; valva nearly parallel-sided, dorsal margin curved distally, costa angled or slightly hooked at distal end; anellus lobes rounded; phallus long and slender, sub-distally with bulge, cornutus near distal end distinct, tapered proximally.

**Female** genitalia (Fig. 9) Papillae anales broad with short setae; apophyses posteriores and anteriores of similar length, apophyses anteriores with basal fork; antrum funnel-shaped with medial ridge; ductus bursae with medial portion sclerotized and curved, anterior portion with two or three coils; corpus bursa with long (490–600 μm) and oval signum; signum with numerous short, transverse ridges.

#### Etymology.

The species’ name, *confusella*, indicates the confusion with its sister species, *B.pinicolella*.

#### Biology.

Due to the confusion of the two species, the biology is insufficiently known. *Batrachedraconfusella* sp. nov. is the well-known species affecting *Pinus*, and it has been found in several localities of pure pine forests. However, a female beaten from an artificial afforestation of *Larix* at a lowland locality in Switzerland without *Pinus* in the nearby surroundings (Bryner in litt.) also belongs to *B.confusella* sp. nov., indicating that *Larix* may be an additional host-plant. *Batrachedrapinicolella*, in contrast, seems to be restricted to forests of Norway spruce (*Piceaabies*), which is considered to be the host-plant. This hypothesis is proved by a dissected Finnish female specimen bred from *Picea*.

Bolov and Sinev (1990) recorded outbreaks of *B.pinicolella* on *Piceaorientalis*, *P.abies*, *P.pungens*, *Abies* sp. (“European”), *A.nordmanniana*, and less frequently on *Pinus* sp. (“eastern”), *P.silvestris*, and *P.pityusa* from the Kabardino-Balkaria republic in northern Caucasus. Possibly both species were involved.

#### Verified specimens of

***Batrachedrapinicolella*.** Finland 1♂ PPe, Oulu; 7.vii.2011; M. Mutanen leg.; L. Aarvik prep. 2015.024; BOLD sample ID: MM21054; ZMUO. 1♀ PPe, Oulu; 7.vii.2011; M. Mutanen leg.; L. Aarvik prep. 2016.001; BOLD sample ID: MM21052; ZMUO. 1♀ A, Eckerö; 12.vii.2007; M. Mutanen leg.; L. Aarvik prep. 2016.002; BOLD sample ID: MM06705; ZMUO. 1♀ St, Rauma; la. *Piceaabies*; 5.iv.2019; J. Itämies leg.; L. Aarvik prep. 2021.01; ZMUO.

Norway 1♀ AAI, Bygland: Heddevika; 29.vii.2008; K. Berggren leg.; BOLD sample ID: NHMO-DAR-5194; KBE. 1♂ AAY, Birkenes: Nordåsen; vii–viii.2016; S. Svendsen leg.; KBE prep. 13526; KBE. 1♂ AAY, Birkenes: Bjorvand; 30.vii.2002; K. Berggren & S. Svendsen leg.; KBE prep. 13510; KBE. 1♀ AAY, Lillesand: Kjøstvedt; 9.vii.2007; K. Berggren leg.; KBE prep. 13514; KBE. 1♂ AK, Bærum: Isi; 11.vii.2003; P. Seglen leg; KBE prep. 13525; KBE. 1♂ AK, Bærum: Sandvika; 20.vii.1928; E. Barca leg.; NHMO prep. 2834; NHMO. 1♂ AK, Ås: Ås; 1.viii.1982; L. Aarvik leg.; NHMO prep. 3900; NHMO. 1♂ AK, Ås: Ås; 11.vii.1983; L. Aarvik leg.; NHMO prep. 3892; NHMO. 1♂ OS, Nordre Land: Tranligrenda; 5.vii.2010; K. Berggren leg.; KBE prep. 13519; KBE. 1♂ VAY, Flekkefjord: Helle; 18.vii.2013; K. Berggren leg.; BOLD sample ID: NHMO-DAR-4109; KBE prep. 13504; KBE. 1♂ VAY, Kristiansand: Gimle Gård; 11.vii.2003; K. Berggren leg.; BOLD sample ID: NHMO-DAR-5193 (failed); KBE prep. 13523; KBE, 1♀ VAY, Kristiansand: Kuholmen; 17.vii.1970; K. Berggren leg.; KBE prep. 13511; KBE. 1♂ VAY, Kristiansand: Nedre Timenes; 31.vii.2011; K. Berggren leg.; BOLD sample ID: NHMO-DAR-4108; KBE prep. 13503; KBE. 1♂ VAY, Kristiansand: Stokken; 8.vii.1978; K. Berggren leg.; KBE prep. 13522; KBE. 1♂ VAY, Kristiansand: Søgne, Torvesanden; 27.vi.2014; K. Berggren leg.; KBE prep. 13517; KBE. 1♂ VE, Larvik: Frydenlund; 12.vii.2013; K. Berggren leg.; KBE prep. 13518; KBE. 1♀ Ø, Fredrikstad: Onsøy, Rauer; 21.vii.1920; E. Barca leg.; NHMO prep. 3961; NHMO. 1♂ Ø, Moss: Rygge, Sildebauen; 10.vii.1981; L. Aarvik leg.; NHMO prep. 3891; NHMO.

Switzerland 1♀, BE, La Neuveville, Ligeresse, 810 m, 4.vii.2002, Larix decidua; leg. R. Bryner; coll. R. Bryner.

Austria 1♂ Vorarlberg, Gaschurn-Partenen, Schuttfluren Lifinar, 1150 m, 31.vii.2018; P. Huemer leg.; BOLD sample ID: TLMF Lep 26889; TLMF. 1♂ Vorarlberg, Gaschurn-Partenen, Schuttfluren Lifinar, 1150 m, 2.vii.2018; P. Huemer leg.; GEL 1275 P. Huemer; TLMF. 1♀ Vorarlberg, Gaschurn-Partenen, u. Ganifer-Schrofen, 1460–1500 m, 19.vii.2019; P. Huemer leg.; GEL 1282 P. Huemer; TLMF. 1♀ Oberösterreich, Nationalpark Kalkalpen, Lackerbodenstraße, 653 m, 14.vi.2009; J. Wimmer leg.; GEL 1287 P. Huemer; TLMF.

Germany 1♀ Baden-Württemberg, Schwarzwald, Buchenberg, 21.vii.1954; H.G. Amsel leg; genitalia in gylcerine; TLMF.

Italy 1♂ Südtirol, Oberrasen, Biotop Rasner Möser S, 1100 m, 5.vii.2015, P. Huemer leg.; DNA Barcode ID TLMF Lep 17972; TLMF.

#### Distribution.

The species pair *B.confusella* sp. nov. and *B.pinicolella* is widely distributed in Europe but seems to be absent from the Mediterranean (http://www.faunaeur.org; accessed on 25.iv.2021). However, as former records have been summarized among the latter taxon, a detailed study of distribution of both species is required for future studies. From our sequenced and/or genitalized material, *B.confusella* sp. nov. together with its major hostplant, *Pinussylvestris*, is distributed in the temperate zones between the Alps in the South and Fennoscandia in the north. *Batrachedrapinicolella* in concordance with its major host-plant, *Piceaabies*, shows a boreo-montane distribution pattern with isolated records from the Alps and mountainous areas of Central Europe as well as northern and north-western Europe. A barcoded specimen of *B.confusella* sp. nov. from Armenia (coll. ZMUC) confirms the presence of this species in Caucasus. According to images of genitalia on the mothdissection.co.uk website, both species are present in the United Kingdom (https://mothdissection.co.uk/species.php?Tx=Batrachedra_pinicolella; accessed on 30.iv.2021)

## ﻿Discussion

Because of their external similarity, the two species have until present been confused. This is in spite of the fact that they have different host plants. Specimens collected in localities with *Pinus* (and no *Picea* present) belong to *B.confusella* sp. n., i.e. from Byrum Sandvik, Öland in Sweden, where only *Pinus* is present and from Belteviga and Bråvann, Kristiansand, Norway. In Finland and Austria, *B.pinicolella* has been collected in places where only *Picea* grows but rarely both species have been found in syntopy. Further breeding experiments are needed to confirm additional hostplants, e.g., in the genera *Abies* and *Larix*. The issue dealt with in the present paper is not unique. DNA barcoding has revealed that there still exist numerous taxonomic problems in the European lepidopteran fauna; see for instance Huemer et al. (2020) concerning Gelechiidae and Lopez-Vaamonde et al. (2021) concerning Gracillariidae. To deal with this situation, systematic and targeted collecting should be encouraged to make material available for taxonomic revisions.

## Supplementary Material

XML Treatment for Batrachedra confusella
